# Psychological impact of exceptional response in people with advanced cancer: a qualitative exploration

**DOI:** 10.1007/s11764-024-01655-7

**Published:** 2024-08-14

**Authors:** Sakeenah Wahab, Anthony Joshua, Haryana M. Dhillon, Megan Barnet

**Affiliations:** 1 Hornsby Ku-Ring-Gai Hospital, Hornsby, NSW Australia; 2 Vincent’s Hospital Sydney, Darlinghurst, NSW Australia; 3 Psycho-Oncology Cooperative Research Group, School of Psychology, Faculty of Science, The University of Sydney, Sydney, NSW Australia; 4 St Vincent’s Hospital Sydney, University of NSW, Sydney, Australia; 5 School of Biomedical Engineering, UTS, Sydney, Australia; 6 Kinghorn Cancer Centre, St Vincent’s Hospital, Darlinghurst, NSW 2010 Australia

**Keywords:** Cancer survivor, Advanced cancer, Exceptional responder, Qualitative study, Adjustment, Psychological support

## Abstract

**Background:**

In the cancer context, exceptional response incorporates unusual or unexpected response to anti-cancer treatment. For this study, exceptionally ‘good’ responses are defined as progression-free survival of more than three times the median from comparable trials. We aimed to explore how people meeting the definition of exceptional response to systemic cancer treatment experience adjust to their unexpected survivorship.

**Methods:**

Individuals with ‘exceptional response’ to anti-cancer therapy nationally were referred by their treating clinicians to the Exceptional Responders Program. We conducted a qualitative sub-study involving semi-structured interviews with purposively selected participants. Those eligible had metastatic cancer, had survived *at least* 3 times the expected time since diagnosis, spoke English, and were aged > 18 years. Interviews were audiorecorded, transcribed and analysed thematically; and continued until thematic saturation was achieved.

**Results:**

Twenty participants were interviewed. Thirteen were male (65%) with a median age of 63 years. Median time since cancer diagnosis was 6.5 years (range 3–18); survival times ranged between 3 and 10 times that expected. We identified four themes which varied in importance between individuals and over time.

**Conclusion:**

Exceptional responders may benefit from routine screening of distress and unmet needs to provide psychosocial support. Clinical services must focus on first capturing and then tailoring care to meet the diverse needs of this growing cohort.

**Implications for cancer survivors:**

Adjustment to a diagnosis of advanced cancer and subsequent unexpected long-term survival is an often isolating experience and is common amongst exceptional responders. Seeking psychological and social support may assist with adjustment.

**Supplementary Information:**

The online version contains supplementary material available at 10.1007/s11764-024-01655-7.

## Introduction

### Psychology of cancer survival

Adjustment to living with cancer is a leading theme in clinical cancer research in recent years. We have learned that carrying the baton as a cancer ‘survivor’ can be an arduous task, and chronic care models require adjustment to address the needs of people living long term with an advanced cancer diagnosis [1, 2]. Cancer diagnosis and treatment, and their aftermath, can cause profound and long-lasting distress for patients, regardless of cancer outcomes. Known sources of distress for cancer survivors include fear of cancer recurrence or progression, general anxiety, physical dysfunction, and persistent neurocognitive impairment [1–5].

Cancer diagnoses may be perceived as traumatic events, particularly when the diagnosis is accompanied by a poor prognosis [6]. Factors influencing an individual’s response to a cancer diagnosis include educational background, personality, socioeconomic status, available support networks, and mental health background [5, 7]. These factors collectively contribute to ‘mindset’, an individual’s core assumptions about how things work in the world that influence how they interpret and respond to situations [8, 9]. Importantly, mindset can change and evolve over time [8], and ‘post-traumatic growth’, for example through strengthening individual resilience, is associated with positive psychological outcomes [3].

Many cancer-specific factors influencing mindset are inherently disrupted for patients with unexpected survival following terminal diagnoses. This includes trust in their healthcare team, which may be significantly undermined by (retrospectively) inaccurate baseline discussions around prognosis. Loss of trust is further confounded by a paucity of data to guide discussions around the likely future trajectory for extreme survival outliers, leaving clinicians, and patients in ‘evidence-free’ zones. While the possibility of a durable response provides hope for patients, particularly those receiving immunotherapy, it remains sufficiently rare, and predictive biomarkers sufficiently poor, that healthcare teams must prepare patients for the likely outcome of cancer progression and death. The minority who ultimately do have a durable response can then be left in a state of limbo—unsure of their role in family, work, and society, and uncertain how they should plan for the future.

Given the dramatic changes in cancer outcomes with the emergence of immunotherapies and targeted treatment, there is a need to understand how those surviving an advanced cancer diagnosis adjust to, what may be, unexpected survivorship.

### Psychological impact of exceptional response

In the Exceptional Responders Program (ExRes), we have collected biospecimens and data from people experiencing an ‘exceptional response’ to anti-cancer therapy. Exceptional response incorporates unusual or unexpected response to anti-cancer treatment (of any form). For this study, exceptionally ‘good’ responses are defined as progression-free survival of more than three times the median from comparable trials [10]. Treating oncologists refer potential cases to ExRes, patients are then contacted for centralised consent via telehealth prior to the collection of clinical details. An independent steering committee reviews cases, and, if approved, peripheral blood is collected locally and couriered to the Garvan Institute for fractionation and biobanking. Here, we aimed to explore how people meeting the definition of exceptional response to systemic cancer treatment experience and adjust to their unexpected survivorship.

## Methods

We conducted an inductive qualitative study using thematic analysis. Centralised Human Research Ethics Committee approval (HREC/18/SVH/171) was obtained for the sub-study as an amendment to the larger project, and all participants provided informed consent.

### Study population

Potentially eligible participants were confirmed as meeting the pre-specified criteria for ‘Exceptional Response’, defined here as receiving systemic treatment (of any kind) and surviving without disease progression for more than three times the median period from comparable trials.

### Procedure

Participants within ExRes meeting our inclusion criteria and who had provided consent to be contacted about future research were invited via email or telephone call to participate in this sub-study (*n* = 30). We purposively sampled to ensure diversity of gender, age, and tumour and treatment type of the eligible participants; 30 individuals were invited to take part. Potential participants were sent the participant information statement and consent form and could choose to return this via email or contact the researchers via telephone. Once written consent was obtained, an interview was scheduled.

### Data collection

Qualitative interviews were conducted by SW and HD. Clinical and demographic information was obtained from the ExRes database or directly from participants. Semi-structured qualitative interviews were conducted via telephone following an interview guide developed by the authors using existing models of cancer survivorship to inform topics for discussion [11]. The interview guide (see Supplementary file 1 for full interview guide) addressed the following areas: diagnosis, treatments and decision-making, pre-existing knowledge, healthcare professional involvement, end-of-life discussions, feelings around survivorship, recovery, and advice. Interviews were conducted as a discussion between the participant alone and the researcher following reflexive practice with researchers adapting questions, probes, and order of interview topics in response to participants. Each participant was interviewed once only. The interviews were audio-recorded, transcribed using Trint, and checked and de-identified by SW.

### Data analysis

The interview transcripts were analysed qualitatively using codebook thematic analysis. Key themes were identified using an inductive approach; SW and HD familiarised themselves with the data and iteratively developed and discussed the codebook themes. Data saturation was reached at 15 interviews and a further five participants were recruited afterwards. The thematic codebook was applied to all transcripts. To ensure rigor and minimise bias, transcripts were reviewed, verbal debriefing after interviews occurred, along with regular discussions within the study team to facilitate bracketing of researcher subjective perspectives. The COREQ checklist was applied to ensure reporting rigor [12] (Supplementary file 3).

### Interviewers

SW was a female, junior doctor at the time of this study from a culturally diverse background. HD was a non-binary, mixed-race, experienced researcher, with more than 15 years of qualitative research practice and substantial involvement in cancer survivorship research and model development. Neither were known to potential participants nor were they involved in the clinical care of participants or ExRes recruitment. Regular debriefing and discussion after interviews provided opportunities to reflect on and bracket any perceived biases. Three interviews were conducted by HD to allow SW to observe the interview process, and HD listened to and provided feedback on the first five interviews conducted independently by SW. Regular discussion and coding comparison during the analysis process provided additional rigor.

## Results

A total of 30 people were invited to participate, with 20 participants interviewed between March 2020 and March 2021. The sample was predominantly male (*n* = 13, 65%) and had a median age of 63 years (range 29–83) (Table 1). Participants had a range of tumour types and previous treatments (see Table 1 for detail). Median time since diagnosis of advanced cancer was 6.5 years (range 3–18), and the mean ‘fold-increase’ ratio of expected to actual survival time was 8.4 (range 3–20.6). The median duration of interviews was 47.5 min (range 29–88).

**Table 1:**
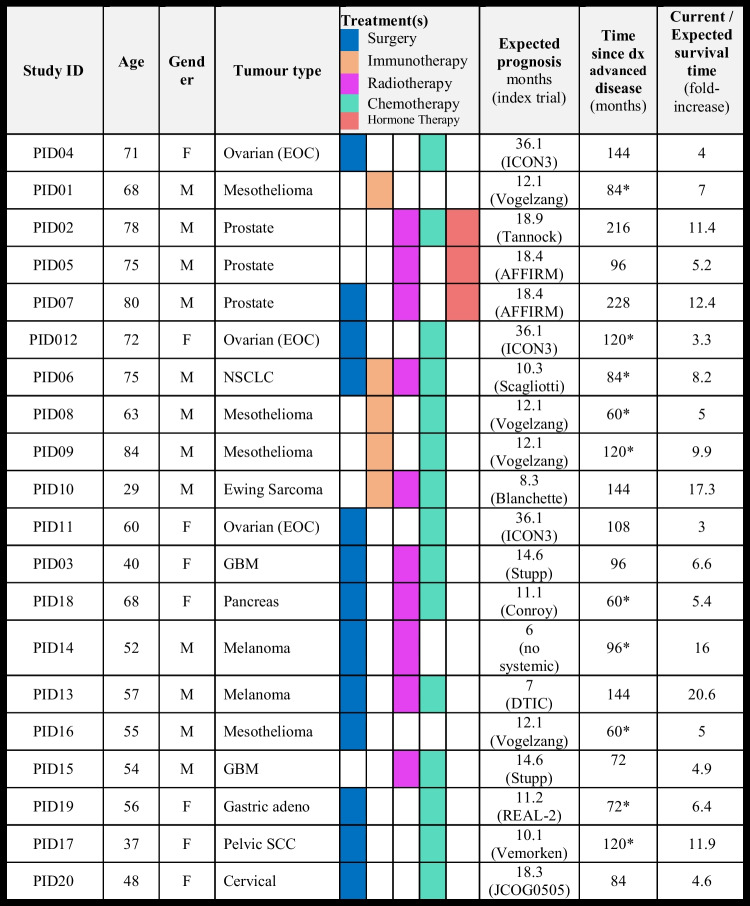
Patient characteristics, treatment types, and expected and observed survival times with individual and mean fold-increase

*Participants with de novo advanced disease

### Exceptional survivorship themes

We identified four themes from interviews with participants: uncertainty, adjustment/adaptation, spiritual/psychological, and social supports. The importance of themes varied between individuals and over time throughout their survivorship trajectory. Each theme is discussed below, and the coding schema is presented in Supplementary File 2.

#### Uncertainty

Uncertainty of likely treatment outcomes and defining risk of morbidity and mortality early in cancer survivorship were common across all participants. At the time of diagnosis, some patients reported persevering for the investigation of physical symptoms or seeking second opinions which they felt expedited their cancer diagnoses. Participants without previous healthcare experience and/or health concerns were less likely to persist with medical help-seeking and felt regret and uncertainty around how this impacted outcomes.

I was from the country and working in a high-pressure job … wish I would’ve gotten a second opinion instead of ignoring what my body was telling me PID020.

Uncertainty around treatment response, future planning, and cancer progression were prominent in participant accounts of their experience.

The docs couldn’t set a time frame for if or when …I might not make it. PID07.

When participants or their family/friends had prior experience with the healthcare system, they indicated a greater acceptance of the uncertainty of their situation. Being aware of the plan if and/or when the cancer recurs or progresses helped mitigate anxiety around uncertainty: ‘I know roughly what the next steps are’ PID13.

Most participants noted trust in their medical team as an important contributor to their coping with uncertainty. Trust was fostered by an open discussion around ‘knowns and unknowns’ PID10.

Expected prognoses in this population varied greatly, as did their preferences for knowing their prognosis. Some patients preferred not to discuss median expected survival with their clinician and were ‘happy for it to be unknown’ PID11. While others used discussion of prognosis to bolster a positive mindset: ‘no reason you can’t be the percentage of positive outcome’ PID03; or, to support their pragmatism: ‘frank/honest conversations were easier than not knowing’ PID06/PID08, respectively*.*

Participants reflected that, from the outset of their cancer experience, the question of mortality was ‘on my mind’ PID03 but was discussed with their clinical teams ‘only when I asked’ PID03*.* Some patients directly asked about the risk of death, to which clinicians tended to respond with statistics. Other patients chose to avoid this discussion, preferring not to define this risk: ‘let’s see what can be done or cannot be done’ PID06. Despite wanting to discuss death, most felt it was not helpful to ‘have a timeline hanging over my head’ PID03, preferring to ‘take it day by day’ PID13 and PID18.

#### Adjustment and adaptation

Constructive acceptance (‘prepare for the worst, hope for the best’ PID01, PID19) and adjustment to a cancer diagnosis were facilitated by awareness of available resources. Engaging with multiple supports; ‘my GP, the community nurses, physiotherapist, support groups’ PID04, PID10 facilitated the evolution of a ‘new normal’ PID10, for some. Others described their emotional experiences as ‘stages of grief’ PID18, in this case despite multi-level support and favourable treatment responses.

I was in shock then felt lost… then out of control. PID12.

I was obviously shocked but eventually had to let go …it felt extremely lonely. PID20.

Older patients (> 50 years) found addressing loose ends such as writing a will or advanced care directive, and discussing important family decisions and plans for financial security helped their adjustment. Younger patients (< 50 years) while initially shocked by the diagnosis, tended to more quickly move on to optimism, particularly focused on future availability of new and better treatment options. Questions about future planning and addressing the logistics of death came later in the treatment pathway for younger participants, often after early lines of treatments stopped working.

Those diagnosed while in regular employment found that workplace transition from ‘well-person’ to ‘cancer patient’ and then ‘cancer patient …without treatment’ PID18 and PID20 was particularly challenging. These perceived identities reflected the difficulties navigating general community perceptions of cancer as a disease which is either present and requiring treatment or cured, whereas participants experienced it as an ‘invisible illness when you don’t look sick’ PID20.

Time heals *most* wounds. Most participants interviewed had moved forward with life ‘alongside’ their diagnoses, with lifestyle modifications ‘I exercise and eat healthy every day’ PID16, social adjustments ‘I talk about it [the cancer] if people ask about it …but that’s it’ PID03, and for some, an increased appreciation for life; ‘…second chance at life, don’t waste it’ PID08*.*

Adjustment and adaptation to the diagnosis of cancer appeared relatively more difficult for exceptional responders with persistent physical symptoms resulting from the disease and/or its treatment; ‘I’m still breathless all the time’ PID06. This was not only because of physical impairment, but because the symptom served as ‘somewhat of a reminder’ PID09, of the diagnosis*.* For these participants, adjustment to the diagnosis of cancer involved recognition that survival comes at a price; ‘some fights you cannot win’ PID14*.*

#### Spiritual/psychological

Despite significant psychological adjustment needs, professional psychology services were reportedly offered to only about one-third of patients by their oncologist or general practitioner combined. Perceptions of the central coordinating health professional through ‘survivorship’ were unclear; ‘…maybe my GP?’, ‘my oncologist? …not really’ PID13*.* The coordinating professional was more often seen as the medical oncologist during active treatment, without a clear point of transition from specialist care to general practice following that. Ongoing specialist involvement was not only perceived as most important for recurrence/relapse monitoring, but also served as a ‘yearly reminder’ PID14, and prompted ‘scan/test anxiety’ PID14*.* Ongoing psychological burden was not addressed in routine oncology follow-up consultations and additional supports were not discussed. Palliative care was discussed with some older participants at the time of diagnosis or first progression, particularly those with long-standing co-morbidities, but was not accepted by most, citing perceived association of the service with death; ‘…means I’m going to die’ PID02 and PID03, ‘felt I’m not ready for that just yet’ PID03*.*

Overarching patterns of psychological adjustment included shock at diagnosis followed by the emergence of hope, anxious anticipation of routine reviews for recurrence/progression, and normalisation of a life incorporating cancer (discussion, investigations, appointments). Many participants self-initiated novel exploration of meaning-making in the form of support groups, public speaking, or bespoke approaches; ‘did my own research’ PID07*.* The majority indicated some degree of increased spiritual/psychological awareness with a uniformly positive impact, particularly around the perception of self-empowerment.

Mindfulness activities, including religious ceremonies, exercise, and meditation, were consistently referenced as positive behaviours. Most participants reported pre-morbid involvement in spiritual activities; the diagnosis of cancer simply prompted prioritisation of self-care into daily routine. Spiritual activities helped patients ‘deal with what may or may not be’ PID16. A few participants had no perceived spiritual/psychological adjustment, either by choice ‘…didn’t feel like I needed it’ PID18 or pre-existing position against spiritual or psychological engagement ‘I don’t believe in that stuff’ PID13.

#### Social supports

Most interviewees associated social support, predominantly comprising friends and family, with improved hope and optimism throughout the disease trajectory. Around half of the participants remembered the ‘significant other’ who was with them at the time of diagnosis, with consistent recollection of how their supporter responded; ‘my husband cried more than me’ PID03, or assisted them; ‘…asked questions and wrote down information for me’ PID13 and PID17. Most reported feeling immediate concern about the impact of the diagnosis on family and friends.

The large majority of participants relied on one or more forms of social support. These included family, friends, religious/spiritual support groups, and pets. The establishment of a new sense of ‘normalcy’ and ‘control’ was greatly influenced by supportive communities in daily life including the workforce; ‘[my workplace was] accepting that I had to take things slower’ PID14*.* Some participants engaged with allied health services in the community, including specialist nurses and rehabilitation programmes, finding in them a source of ‘comfort and support’ PID20.

Loneliness was reported by some during both active treatment and survivorship. Participants with little previous healthcare experience were particularly at risk of loneliness. When participants had perceived mental health deterioration, they most often sought help from psychiatrists and psychologists. Other supports identified at times of need included support groups, educational activities, and accepting new challenges, such as public speaking.

## Discussion

Treatment breakthroughs, such as immunotherapy, are significantly changing prognosis for *some* people, but notably creating a prognostic dichotomy of *durable responders* (the minority, whose survival far exceeds that predicted) and *non-responders* (the majority, in whom baseline discussions of prognosis are accurate or overestimates). The increasing number of people living beyond their projected prognoses has highlighted a need for greater access to tailored cancer survivorship care. People are ‘…considered a cancer survivor from the time of diagnosis through the balance of life. There are many types of survivors, including those living with cancer and those free of cancer’ [7]. While this definition includes those living with cancer, support for cancer survivors has largely focused on people with earlier-stage disease treated with curative intent. While survivorship care and rehabilitation are part of the trajectory of quality cancer care, exceptional responders’ access to these forms of care is often lacking as they sit in a liminal space with a poor prognosis cancer diagnosis and a tumour response, but outside the survivorship care frameworks. As our results demonstrate, there are important gaps in survivorship support for this population, who carry significant uncertainty about their disease trajectory.

Our interviews with exceptional responders to cancer treatments identified four themes: uncertainty, adjustment/adaptation, spiritual/psychological, and social supports. Uncertainty was commonly expressed and varied across individuals and the disease trajectory. Having direct conversations with a trusted healthcare provider mitigated fear and uncertainty associated with cancer recurrence, consistent with previous literature [13, 14]. Notably, omission of such direct conversations may be purposeful, due to clinical concern for a patient’s psychological well-being or a clinician’s own uncertainty or time-restraints, or accidental, due to focus on other concerns. Uncertainty was a less prominent theme for younger patients; this may relate to less exposure to death and bereavement, fewer co-morbidities, and greater pre-morbid life expectancy in this population [2].

Uncertainty around long-term prognosis and disease outcomes for exceptional responders presents a clear challenge to their everyday lives. Risk-stratified survivorship estimation is currently hampered by the lack of dynamic predictive and prognostic biomarkers, making discussions around likely outcomes for this cohort ‘evidence-free’. There is a critical need to incorporate long-term survivors into biomarker research to help define risk-stratification algorithms over time. Concurrent with this, it is critical that discussions between survivors and healthcare professionals around areas of uncertainty, specifically prognosis and future planning, are tailored to individual preferences about information and regularly re-visited.

Frank conversations and opportunities to ask questions of their healthcare providers help survivors to (1) establish realistic expectations (shared by the individual and healthcare professionals) and (2) live ‘alongside’ uncertainty, rather than feeling overwhelmed or fearful. Actual risk of recurrence does not necessarily predict fear of recurrence in cancer survivors, but an individual’s *appraisal* of this risk may differ significantly from the *actual* risk. A recent Dutch study identified cancer recurrence risk *appraisal* (rather than objective risk) as the most predictive of fear of cancer recurrence [15]. Regular assessment of a patient’s beliefs around risk of recurrence or progression may reduce anxiety, or at least prevent its escalation. Those with enduring high levels of fear of cancer recurrence may benefit from evidence-based psychological interventions, such as conquer fear or fear of cancer recurrence therapy [4]. While uncertainty cannot be removed, its impact on psychological well-being can be managed with careful discussion and regular review.

Life stage appears to impact adjustment to an uncertain prognosis. In our population, older survivors described greater acceptance of a terminal diagnosis, facilitated by planning for end of life and tying up loose ends. Supporting older survivors in communicating with family and friends about their wishes may assist them in adjusting to their uncertain prognosis. While younger patients, particularly those in regular employment may need assistance navigating their social and professional roles.

Post-traumatic growth across the spiritual dimension, when endorsed by individuals, is generally beneficial to psychological well-being and associated with improved resilience [16]. For many in our cohort, spirituality through mindfulness was identified as a core component of self-care with a positive impact on psychological well-being. Some without pre-existing spiritual beliefs, however, built meaning and resilience using other strategies. One striking feature of our results is the limited engagement with psycho-oncology and peer-support services, suggesting many exceptional responders are navigating their adjustment to illness without professional support.

The loneliness of living with cancer struck many of our participants at some point during treatment or recovery, particularly those with minimal prior experience of the healthcare system. One strategy used to ameliorate loneliness was peer support. Peer support can be defined as social support provided by other individuals with the same disease, based on their knowledge and lived experience. It may take the form of individual ‘one-to-one’ connections or group-based support. The evidence for the efficacy of peer support programmes is mixed, largely as a result of studies assessing differing endpoints. One systematic review of peer support found weak to moderate association with improved components of psychological empowerment [16]. Another focused on survivor outcomes including quality of life, depression/anxiety, and self-efficacy, reporting significant improvements in all endpoints [17]. Regardless, active engagement in new activities (including those involving peer support) indicates post-traumatic growth and is associated with higher levels of resilience and lower levels of distress [11]. Peer support, when conducted with trained leaders, is a low-cost intervention with the potential for improvement in the adjustment and experience of people living with cancer. One under-explored outcome for peer support is the impact on survivor loneliness; this should be included as an endpoint in future studies.

It is clear from the mixed experience of our participants that access to survivorship care and support remains inconsistent across cancer services, stages, and tumour types. The Clinical Oncological Society of Australia (COSA) has previously published a model of cancer survivorship care suggesting integration from point of diagnosis to risk-stratified follow-up care involving all sectors of the healthcare system is required to best meet the needs of cancer survivors [11]. The experience of our participants illuminates the need for better integrated survivorship care for those living with advanced or metastatic disease, particularly as the population of exceptional responders continues to grow.

A strength of our study was the inclusion of people with lengthy exceptional responses to treatment, drawing on their direct experience of living long-term with an uncertain prognosis. We used rigorous methodology to conduct this qualitative analysis and study, with multiple interviewers, coders, and direct use of participant quotes. A key limitation of the study is the potential bias amongst willing participants to those with more spiritual or positive experience of cancer survivorship. Additionally, we interviewed participants once only and were not able to demonstrate their personal mindset growth or change although all participants had survived at least 5 years since receiving their poor prognosis which afforded them time to reflect on their mindset change.

## Conclusion

Success in oncology can be measured in many ways, including survival outcomes. By most measures and by their own accounts, durable responders epitomise success in cancer. Those interviewed here demonstrate gratitude for a life they did not think they would have. However, underlying their apparent adjustment to living long-term with a cancer diagnosis remains angst associated with an uncertain prognosis. Clinical services must focus on capturing and tailoring care to meet the needs of this group of survival outliers in survivorship care, ensuring they receive evidence-based support to meet their diverse needs.

## Supplementary Information

Below is the link to the electronic supplementary material.

Supplementary file1 (DOCX 25 KB)

Supplementary file2 (DOCX 16 KB)

Supplementary file3 (DOCX 17 KB)

## Data Availability

No datasets were generated or analysed during the current study.
