# Supplementary material for: Psychological impact of exceptional response in people with advanced cancer: a qualitative exploration
Source: J Cancer Surviv. 2024 Aug 14;20(2):361–8. doi: 10.1007/s11764-024-01655-7 (PMC12988963; doi:10.1007/s11764-024-01655-7)
Supplement: Supplementary file 1 — Supplementary file1 (DOCX 25 KB) [file 11764_2024_1655_MOESM1_ESM.docx]

**Exceptional Responders: Psychological Impact Substudy Patient Interview Schedule**

**Initial exploration (rapport building)**

Could you tell me about how you were diagnosed with cancer? (What type?)

Probe for: which medical specialties were involved in the diagnosis, setting of initial lx/Mx How did you feel when you were diagnosed?

What did you think this diagnosis meant for your future? Was prognosis discussed? Who with?

Was there someone with you at the time you were told about your diagnosis? Have they been involved in your treatment and care since your diagnosis?

**Treatment & decision-making**

Do you recall having to make decisions about treatment? How did you go about making those decisions?

Probe with: involving family & friends, medical specialist advice (who and how), GP. How did you feel about these decisions?

What plans did you change for the future? What plans did you make for the future? What plans do you have now for the future?

**Pre-existing personal biases**

What did you know about your cancer before you were diagnosed?

Has your experience since your diagnosis been the same or different to how you expected things would be? How has it been the same? How has it been different?

**Health professionals involved in care**

Was there a main medical specialist managing your care since your diagnosis?

If so whom and how long

Otherwise outline and comment on the differences on journey (if any) How has your General Practitioner been involved in your care?

Did you see a physician? How were they involved in your care? Did you see a surgeon? How were they involved in your care?

Did you see a medical oncologist? How were they involved in your care? Did you see a radiation oncologist? How were they involved in your care?

Did you see a palliative care physician? How were they involved in your care? Did you see a community nurse? How were they involved in your care?

**End of life/mortality discussions**

When were End of Life discussions brought up?

Probe: Who brought it up and/or support network(s) available if utilized Did you ever have a discussion about an advanced care directive?

Were they revisited at any time? (If so, when?)

Probe: ?Psychologist and/or Psychiatrist/ mental health Do you have any spiritual/ religious beliefs? •

What was good about your experience?• What was bad about your experience?

Exceptional Responders: Psychological Impact Substudy Patient Interview Schedule v1.0 dated 13/4/20 .

. Page 1 of 2

**Feelings around survivorship**

What are the dominant feelings you experience now about the cancer?

Probe: About your current health? Around the time of scans? Discussing with family members or friends?

How often do you think about the cancer? How does that change with time?

Probe: After 2 years? After 5 years?

If this has changed with time, why?

Do you prefer to talk about the cancer or to avoid talking about the cancer with family? Friends? Did you access any rehabilitationservices?

Physio, exercise support, pulmonary rehab, cardiac rehab, survivorship groups, support groups

- Cancer Council support services (13 1120)?

**Advice**

Are there things that could have been done differently that may have improved your experience?

What would you recommend to other people diagnosed with cancer in the future?

What would you recommend clinicians to do in their discussions with cancer patients in the future?

What other services do you feel would have helped you?

\

Exceptional Responders: Psychological Impact Substudy Patient Interview Schedule v1.0 dated 13/4/20

Page 2 of 2
