# Supplementary material for: Psychological impact of exceptional response in people with advanced cancer: a qualitative exploration
Source: J Cancer Surviv. 2024 Aug 14;20(2):361–8. doi: 10.1007/s11764-024-01655-7 (PMC12988963; doi:10.1007/s11764-024-01655-7)
Supplement: Supplementary file 2 — Supplementary file2 (DOCX 16 KB) [file 11764_2024_1655_MOESM2_ESM.docx]

**Exceptional Responder Substudy**

**Coding schema**

1. Uncertainty:
   1. Uncertainty of treatment outcomes and morbidity
   2. Impact of route to diagnosis
      1. Persistence to reach diagnosis
      2. Prior healthcare system experience
   3. Prognosis:
      1. desire to know or not
      2. Regardless, what can be done?
      3. Mortality awareness
2. Adjustment/adaptation:
   1. Acceptance and adjustment
   2. Aids to adjustment
   3. Grieving process
   4. Dealing with practical matters
   5. Workplace adjustment
   6. Invisible illness, symptoms
3. Spiritual/psychological:
   1. Care services:
      1. Psychological support
      2. Coordinator of care
      3. Palliative care
   2. Mindfulness activities
      1. Spiritual
      2. Non-spiritual
4. Social supports
   1. Spouse, family, friends
   2. Community support
   3. Healthcare professional support
