# Supplementary material for: Psychological impact of exceptional response in people with advanced cancer: a qualitative exploration
Source: J Cancer Surviv. 2024 Aug 14;20(2):361–8. doi: 10.1007/s11764-024-01655-7 (PMC12988963; doi:10.1007/s11764-024-01655-7)
Supplement: Supplementary file 3 — Supplementary file3 (DOCX 17 KB) [file 11764_2024_1655_MOESM3_ESM.docx]

| **Item** | **Question/topic** | **Page number** |
| --- | --- | --- |
| **Domain 1: Research team and reflexivity** | | |
| **Personal Characteristics** | | |
| 1 | Interviewer/facilitator  Which author/s conducted the interview or focus group? | Line 78, page 3 |
| 2 | Credentials  What were the researcher’s credentials? E.g. PhD, MD | Title page |
| 3 | Occupation  What was their occupation at the time of the study? | Title page |
| 4 | Gender  Was the researcher male or female? | 4 |
| 5 | Experience and training  What experience or training did the researcher have? | 4 |
| **Relationship with participants** | | |
| 6 | Relationship established  Was a relationship established prior to study commencement? | 4 |
| 7 | Participant knowledge of the interviewer  What did the participants know about the researcher? e.g. personal goals, reasons for doing the research | 4 |
| 8 | Interviewer characteristics  What characteristics were reported about the interviewer/facilitator? e.g. Bias, assumptions, reasons and interests in the research topic | 4 |
| **Domain 2: study design** | | |
| **Theoretical framework** | | |
| 9 | Methodological orientation and Theory  What methodological orientation was stated to underpin the study? e.g. grounded theory, discourse analysis, ethnography, phenomenology, content analysis | 3 |
| **Participant selection** | | |
| 10 | Sampling  How were participants selected? e.g. purposive, convenience, consecutive, snowball | 3 |
| 11 | Method of approach  How were participants approached? e.g. face-to-face, telephone, mail, email | 3 |
| 12 | Sample size How many participants were in the study? | 4 |
| 13 | Non-participation How many people refused to participate or dropped out? Reasons? | N/A |
| **Setting** | | |
| 14 | Setting of data collection Where was the data collected? e.g. home, clinic, workplace | 3 |
| 15 | Presence of non-participants Was anyone else present besides the participants and researchers? | 3 |
| 16 | Description of sample What are the important characteristics of the sample? e.g. demographic data, date | 4 & Table 1 |
| **Data collection** | | |
| 17 | Interview guide Were questions, prompts, guides provided by the authors? Was it pilot tested? | Supp File 1 |
| 18 | Repeat interviews Were repeat interviews carried out? If yes, how many? | 3 |
| 19 | Audio/visual recording Did the research use audio or visual recording to collect the data | 3 |
| 20 | Field notes Were field notes made during and/or after the interview or focus group? | 3 |
| 21 | Duration What was the duration of the interviews or focus group? | 4 |
| 22 | Data saturation Was data saturation discussed? | 4 |
| 23 | Transcripts returned Were transcripts returned to participants for comment and/or correction? | N/R |
| **Domain 3: analysis and findings** | | |
| **Data analysis** | | |
| 24 | Number of data coders How many data coders coded the data? | 4 |
| 25 | Description of the coding tree Did authors provide a description of the coding tree? | 4 |
| 26 | Derivation of themes Were themes identified in advance or derived from the data? | 3 |
| 27 | Software What software, if applicable, was used to manage the data? | N/R |
| 28 | Participant checking Did participants provide feedback on the findings? | N/R |
| **Reporting** | | |
| 29 | Quotations presented Were participant quotations presented to illustrate the themes / findings?  Was each quotation identified? e.g. participant number | 6-9 |
| 30 | Data and findings consistent Was there consistency between the data presented and the findings? | 6-9  Supp file 2 |
| 31 | Clarity of major themes Were major themes clearly presented in the findings? | 6-9  Supp file 2 |
| 32 | Clarity of minor themes Is there a description of diverse cases or discussion of minor themes? | 6-9  Supp file 2 |
